# An alternative model for assessing mortality risk in Stevens Johnson syndrome/toxic epidermal necrolysis using a random forests classifier: A pilot study

**DOI:** 10.3389/fmed.2022.935408

**Published:** 2022-12-08

**Authors:** Omar Shareef, James T. Kwan, Sarina Lau, Mohammad Ali Tahboub, Hajirah N. Saeed

**Affiliations:** ^1^Harvard College, Cambridge, MA, United States; ^2^Department of Ophthalmology, Massachusetts Eye and Ear Infirmary, Harvard Medical School, Boston, MA, United States; ^3^Tufts University School of Medicine, Boston, MA, United States; ^4^Department of Biology, Simmons University, Boston, MA, United States; ^5^Department of Ophthalmology, Illinois Eye and Ear Infirmary, University of Illinois Chicago, Chicago, IL, United States

**Keywords:** Stevens-Johnson syndrome (SJS) and toxic epidermal necrolysis (TEN) (SJS/TEN), machine learning, random forest (bagging) and machine learning, SCORTEN score, mortality risk, ABCD

## Abstract

**Introduction:**

Mortality risk prediction is an important part of the clinical assessment in the Stevens-Johnson syndrome and toxic epidermal necrolysis (SJS/TEN) patient. The SCORTEN and ABCD-10 scoring systems have been used as predictive clinical tools for assessing this risk. However, some of the metrics required in calculating these scores, such as the total body surface area (TBSA) involvement, are difficult to calculate. In addition, TBSA involvement is calculated in a variety of ways and is observer dependent and subjective. The goal of this study was to develop an alternative method to predict mortality in patients with SJS/TEN.

**Methods:**

Data was split into training and test datasets and preprocessed. Models were trained using five-fold cross validation. Out of several possible candidates, a random forests model was evaluated as being the most robust in predictive power for this dataset. Upon feature selection, a final random forests model was developed which was used for comparison against SCORTEN.

**Results:**

The differences in both accuracy (*p* = 0.324) and area under the receiver operating characteristic curve (AUROC) (*p* = 0.318) between the final random forests model and the SCORTEN and ABCD-10 models were not statistically significant. As such, this alternative method performs similarly to SCORTEN while only requiring simple laboratory tests from the day of admission.

**Discussion:**

This new alternative can make the mortality prediction process more efficient, along with providing a seamless implementation of the patient laboratory tests directly into the model from existing electronic health record (EHR) systems. Once the model was developed, a web application was built to deploy the model which integrates with the Epic EHR system on the Fast Healthcare Interoperability Resources (FHIR) Application Programming Interface (API); this only requires the patient medical record number and a date of the lab tests as parameters. This model ultimately allows clinicians to calculate patient mortality risk with only a few clicks. Further studies are needed for validation of this tool.

## Introduction

Stevens-Johnson syndrome (SJS) and toxic epidermal necrolysis (TEN) are both mucocutaneous diseases that result in blistering and desquamation of the skin and mucous membranes (1, 2). The distinction between SJS and TEN is based upon the amount of skin involvement. Epidermal detachment less than 10% is considered SJS, while that between 10–30% is considered SJS-TEN overlap, and skin involvement greater than 30% is classified as TEN (3). While the exact etiology of SJS/TEN is not well understood, most cases occur as hypersensitivity reactions from the use of certain medications and can be life-threatening (4, 5).

The SCORTEN scoring system was developed in 2000 as a severity-of-illness score for SJS/TEN and has since been used for predicting mortality for individuals with SJS/TEN. The model uses seven independent risk factors to predict mortality. These include age > 40 years, heart rate > 120 beats per minute, cancer/hematologic malignancy, TBSA involvement (TBSAI) at day 1 > 10%, serum urea level > 28 mmol/L, serum bicarbonate level < 20 mmol/L, and serum glucose levels > 14 mmol/L. Presence of these risk factors indicates a higher mortality risk for the patient (6). While still an important parameter clinically, predictors such as TBSAI can be difficult to assess accurately for various reasons including the subjectivity in calculating TBSAI, the various methods that are used to calculate it, and delays in transfer to a burn unit or hospital which manages SJS/TEN patients.

In addition to the SCORTEN scoring system, another risk prediction model known as ABCD-10 has been developed recently for assessing mortality risk in patients with SJS/TEN (7). This model uses five independent risk factors for mortality prediction: age > 50 years, epidermal detachment > 10% TBSA, serum bicarbonate level < 20 mmol/L, cancer malignancy, and ongoing dialysis (7). Cancer malignancy and ongoing dialysis are associated with greater degrees of risk according to the model (7). The ABCD-10 model still requires further validation; however, preliminary studies seem to show that SCORTEN performs similarly or better than the ABCD-10 model (8, 9).

Due to the vast amount of clinical data that can now be collected through electronic health record (EHR) systems, machine learning (ML), and artificial intelligence (AI) techniques have come into greater use recently to improve clinical decision making (10). The random forests classifier is a ML technique that was developed in 2001 and employs a combination of decision tree classifiers to allow for greater generalization and reduced noise (11). The algorithm has been validated in a number of studies including those on early glaucoma detection with spectral-domain optical coherence tomography (12), classification of melanocytic lesions using dermoscopic images (13), breast cancer diagnosis (14), and prediction of stroke outcome (15).

The purpose of this study was to develop an alternative model to SCORTEN which uses laboratory results to serve as a simpler way to predict mortality and that can be easily incorporated into existing EHR systems. Since the random forest classifier has proven useful in other medical contexts, it was a natural choice when seeking to build an alternative to the SCORTEN model.

## Materials and methods

### Obtaining the data

Access to a database of individuals diagnosed with SJS/TEN was obtained (*n* = 452). All data were collected and managed using Research Electronic Data Capture (REDCap). Data collected included demographics (Table 1) and lab values. Lab values relevant to the SCORTEN and ABCD-10 scoring systems (Table 2) were collected as well as 96 labs values unique to this study. Data samples were taken from patients who had an acute SJS/TEN episode that required admission to a Mass General Brigham hospital. All laboratory tests used for training the model were taken upon admission. Patients who did not have laboratory tests upon admission were not included in the study. There were a total of 192 patients who met these criteria. Laboratory test data were collected for these patients which included 96 unique values. The laboratory test data for these 156 patients were then split into training (*n* = 156) and test (*n* = 36) datasets to be used for building and validating the model. Demographic data of both the training and test dataset can be found in Table 1.

**TABLE 1:** Demographics of training and test datasets.

	Training dataset		Testing dataset	
		
Demographics	Survived to discharge (*n* = 129)	Died in the hospital (*n* = 27)	Survived to discharge (*n* = 29)	Died in the hospital (*n* = 7)
Female, no.	71	9	14	5
Age (mean)	46	60	44	66
Hispanic or Latino	6	0	4	0
***Race***				
White	83	15	16	6
Black	18	6	3	0
Asian	10	2	2	0
Other	1	0	0	0
Unknown/Not reported	17	4	8	1

**TABLE 2:** SCORTEN and ABCD scores for testing data.

Testing data	Survived (*n* = 29)	Died (*n* = 7)
**SCORTEN at admission**		
Age > 40	17	6
HR > 120	5	1
BSA > 10%	10	4
Cancer	4	2
Serum BUN > 28 mg/dL	4	3
Serum glucose > 252 mg/dL	1	0
Serum bicarbonate < 20 mmol/L	10	2
Mean SCORTEN score	1.76	2.57
**ABCD at admission**		
Age > 50	14	4
BSA > 10%	10	4
Cancer	4	2
Serum bicarbonate < 20 mmol/L	10	2
Dialysis	1	0
Mean ABCD-10 score	1.55	2.00

### Data pre-processing

Categorical data were split using one-hot encoding, and missing values for categorical data were imputed with the mode. The remaining missing values in the training dataset were handled using *k*-nearest neighbors imputation (*k* = 3).

### Model training

A number of different types of classifiers were initially tested on the dataset using five-fold cross validation. The implementation of all classifiers was carried out using the sci-kit learn Python library. These included support-vector machine (SVM), logistic regression, ridge regression, decision tree, and random forest classifiers (RFC) models. The laboratory data were fed into the model, and the ground truth corresponded to whether the patient had died during the acute SJS/TEN episode or survived. Upon cross validation, it was found that an RFC was the most robust in predictive power and was then used for the remainder of the study. Cross validation was then performed again to find the best hyperparameter set for an RFC model that was trained on the full set of predictors. The top five predictors on the training set of this model were found, and the remaining RFCs were trained using only this subset of predictors. This subset of predictors in order of importance were: nucleated red blood cell (NRBC) number, total bilirubin, prothrombin time (PT), white blood cells (WBC), and red blood cells (RBC). The data were pre-processed again in the same manner as the full dataset with this subset of predictors to ensure past predictors were not affecting the newer model. Because the dataset was quite imbalanced, with there being fewer deceased patients than living patients due to the mortality rate of SJS/TEN, various techniques were used to account for this. RFC models were trained on a dataset with no imbalance correction, upsampling of the data, and downsampling of the data. Imbalance correction was carried out using the imblearn library. Upsampling yielded the RFC model with the greatest accuracy when trained on the subset of predictors.

### Deploying the application

Once the model was trained, it was deployed into a clinician facing web app using Flask and Python along with Epic on FHIR API. By integrating Epic on FHIR API, clinicians can enter the MRN of the patient and the date for the lab tests that they wish to use to input into the model. If relevant lab tests were collected on that date, the model will output a mortality score. The deployment of the model in a web app paired with integration of the Epic on FHIR API makes the use of this mortality prediction system extremely simple.


95% CI=A±1.96SE(A)

### Statistical analysis

Accuracy between the SCORTEN and RFC models were compared using a paired *t*-test. Area under the receiver operating curve (AUROC) were compared using the method outlined by Hanley and McNeil (16). The 95% confidence interval was calculated as follows:


95% CI=A±1.96SE(A)

Where *A* is the AUROC and SE is the standard error of the AUROC. The SE was determined as follows:


$$\begin{array}{l} \text{SE }(\text{AUROC})=\\ \sqrt{\frac{A(1-A)+\left(n_{p}-1\right)\left(Q1-A^{2}\right)+(n_{n}-1)\left(Q2-A^{2}\right)}{Np*Nn}} \end{array}$$

Where *n_p_* is the number of positive cases in the test set (which corresponds to individuals who were deceased after the acute SJS/TEN episode) and *n*_*n*_ is the number of negative test cases in the test set (which denotes the individuals who remained alive).

Q1 and Q2 were determined as follows:


$$Q1=\frac{A}{2-A},\;Q2=\frac{2*A^{2}}{1+A}$$

Where Q1 is the probability that two randomly chosen samples of deceased individuals will both be ranked with greater suspicion than a randomly chosen living individual and Q2 is the probability that one randomly chosen deceased individual will be ranked with greater suspicion than two randomly chosen living individuals.

## Results

**FIGURE 1:**
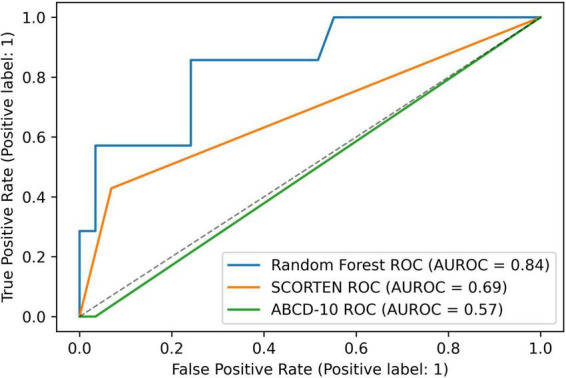
A graph depicting receiver operating characteristic (ROC) curves for random forest, SCORTEN, and ABCD-10 scoring systems.

SCORTEN and ABCD score breakdowns can be found in Table 2. For the testing data, the accuracy, AUROC, specificity, and sensitivity for the SCORTEN model were 0.833, 0.688 (95% CI, 0.45–0.92), 0.931, and 0.429, respectively. For the RFC model, the accuracy, AUROC, specificity, and sensitivity were 0.889, 0.842 (95% CI, 0.65–1.03), 1.0, and 0.429, respectively. For the ABCD model, the accuracy, AUROC, specificity, and sensitivity were 0.778, 0.574 (95% CI, 0.33–0.82), 0.966, and 0.0. These results have been summarized in Tables 3, 4. The differences in both accuracy (*p* = 0.324) and AUROC (*p* = 0.318) were not statistically significant when comparing SCORTEN to the RFC model. Differences in accuracy (0.083) and AUROC (0.091) were not statistically significant for ABCD-10 versus the RFC model as well. The five features used to train this algorithm in order of importance were: NRBC count, total bilirubin, PT, WBC count, and RBC count (LOINC codes: 771-6, 42,719-5, 5,902-2, 6,690-2, and 789-8, respectively). The ROC curves can be seen in Figure 1.

**TABLE 3:** SCORTEN versus random forest performance.

	SCORTEN	Random forest	*P*-value
Accuracy	0.833	0.889	0.324
AUROC	0.688	0.842	0.318
Specificity	0.931	1.000	
Sensitivity	0.429	0.429	

**TABLE 4:** ABCD versus random forest performance.

	ABCD	Random forest	*P*-value
Accuracy	0.778	0.889	0.083
AUROC	0.574	0.842	0.091
Specificity	0.966	1.000	
Sensitivity	0.000	0.429	

**FIGURE 2:**
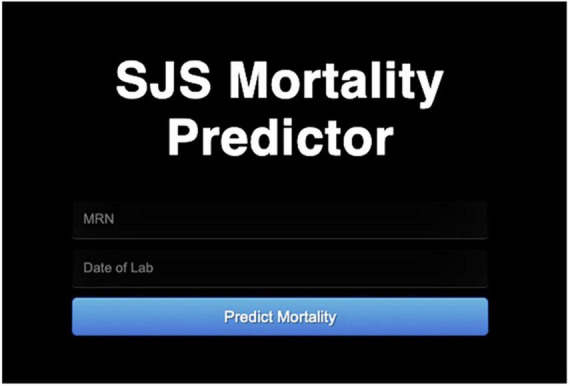
Deployment interface for web application.

The model was then deployed on a web server using the Epic on FHIR API. An example of an integrated SJS/TEN Mortality Predictor is shown in Figure 2.

## Discussion

The medical community has progressed significantly in its understanding of SJS/TEN but mortality rates continue to remain high at 10–34% (3). Accurate assessment of mortality risk is important in disease prognostication, management, and patient/family provider discussions. It is unclear whether systemic treatments beyond supportive care improve mortality, but there has been recent interest in various immunomodulatory therapies in reducing mortality risk (3). Determining the effectiveness of these potential treatments for different risk cohorts requires accurate and precise mortality risk measurement. Patient and family counseling also heavily relies on mortality risk assessment.

This mortality risk is traditionally calculated with the SCORTEN, a prognostic score which predicts in-hospital mortality during acute SJS/TEN. A high SCORTEN score may also be a risk factor for death after discharge (17). SCORTEN is also often used as a benchmark against which mortality after treatments and interventions are compared.

The SCORTEN model uses seven independent risk factors upon admission to predict mortality: age > 40 years, heart rate > 120 beats per minute, cancer/hematologic malignancy, TBSA involvement > 10%, serum urea level > 28 mmol/L, serum bicarbonate level < 20 mmol/L, and serum glucose levels > 14 mmol/L. The most difficult to calculate of these is the TBSA. For SCORTEN, an exact degree of TBSA involvement isn’t required, but rather, an assessment of TBSA involvement as less than or greater than 10%. Values significantly lower or higher than 10% are easily calculated and categorized, but those closer to 10% are difficult to categorize as measurement of TBSAI is highly observer dependent and subjective, and there are a variety of methods used to determine the TBSA involved. Furthermore, a single TBSAI measurement does not take into account progressive epidermal loss and TBSAI may be more sensitive to change over time as compared to other risk variables (17). Other alternatives to SCORTEN have been proposed; the most popular being ABCD-10 which stands for age, bicarbonate, cancer, dialysis, 10% BSA. Studies comparing the accuracy of SCORTEN and ABCD-10 have largely found SCORTEN to be superior or equivalent to ABCD-10 (18–20). ABCD-10, like SCORTEN, also requires calculation of TBSAI.

While TBSAI remains a clinically important tool, particularly in determining fluid resuscitation and management, it may not need to be used as a variable in a mortality risk prediction model as demonstrated by this pilot study. Another model of mortality risk prediction, described in 2021, uses the red cell distribution width to hemoglobin ratio as categorical measurements and found this alone to be comparable to the SCORTEN scoring system (21).

In addition to the difficulty in calculating TBSAI, a common criticism of both the SCORTEN and ABCD-10, is that they simplify continuous and dynamic biologic measurements into dichotomous variables, losing a significant amount of information, particularly in the skin assessment which does not take morphology or location into account (18).

While our proposed RFC model does not account for dynamic biologic measurements over time, the use of several continuous variables that are automatically pulled may allow for greater accuracy in mortality risk prediction, better prognostication over time, and may prove useful as a daily monitoring tool. None of the current scoring systems takes into account the time point after disease onset when a patient was admitted, as all variables are collected within 24 h of admission, with no regard to disease onset. It is unclear how much of an effect admission delay has on the accuracy of scoring systems. A 2006 study found that delay-adjusted SCORTEN scores were comparable to crude scores but that there was significant difference in score between days 1 and 4 (19). Another study showed that SCORTEN and ABCD-10 performed differently depending on when after admission data were collected (20). Our model also does not take time point into account; however, we believe that the use of only lab values as continuous variables may allow for stability over time. We plan to study this in the near future.

In our model, the top five predictors in order of importance were NRBC count, total bilirubin, PT, WBC count, and RBC count. These values from day of admission were the only ones used in the RFC model and are easily calculated and standard of care. The deployment of the model using a web server demonstrates the ease of use when implementing this alternative method. Using the Epic on FHIR API for pulling EHR data from patients in existing healthcare systems, a web app was built that allows clinicians to log in with their Epic credentials. By inputting the MRN of the patient in question and the date of the laboratory tests which they would like to use, the web app is able to use the Epic on FHIR API to pull the necessary laboratory tests and output a predicted probability score for the mortality of the patient using the RFC trained above. Such an application can prove useful in a clinical setting by allowing clinicians to easily, accurately, and quickly predict mortality for patients with SJS/TEN and decide on a course of action for the patient’s treatment. These objective measures can also prove useful in both prospective and retrospective research studying the effect of treatments on mortality. Furthermore, interobserver discrepancies in TBSAI would not affect the calculated mortality risk.

While further validation of this model needs to be done on larger datasets and in a prospective manner, the similar performance between the SCORTEN and our RFC model indicates that the RFC model may be utilized as an alternative to SCORTEN in the future. Furthermore, given that the RFC model relies only on objective data that can be gleaned from patient laboratory tests, the RFC model may be put into use more simply than other methods.

## Limitations

The main limitation in this study was the sample size. The sample size in this study is significant given how rare SJS/TEN is and the single-center nature of this study, but ML models perform best with larger datasets. This pilot study demonstrates the potential utility of this RFC model as a proof of concept but should be tested on larger datasets with multicenter involvement. We plan to conduct these larger studies in the future to further validate and improve the model before implementing it in any clinical settings.

Furthermore, due to disease progression for some SJS/TEN patients, mortality predictions may change as time progresses. Due to the retroactive nature of the data collection, the time points of mortality risk calculation were not standardized. However, most patients had the necessary data to calculate risk at the day of admission so this was the only time point used in this study. Future improvements to the model include factoring in various time points during the patient’s treatment period in a prospective fashion for predicting patient mortality over a longer period of time, including after discharge.

## Data availability statement

The datasets presented in this article are not readily available because they include confidential patient data. Requests to access the datasets should be directed to HS, hnsaeed@uic.edu.

## Author contributions

OS and HS contributed to the conception and design of the study. JK, SL, and MT organized the database. OS performed the statistical analysis. OS wrote the first draft of the manuscript. OS, JK, and HS wrote the sections of the manuscript. All authors contributed to manuscript revision, read, and approved the submitted version.

## References

[1] Mockenhaupt M. The current understanding of Stevens-Johnson syndrome and toxic epidermal necrolysis. *Expert Rev Clin Immunol.* (2011) 7:803–13; quiz 814–5. 10.1586/eci.11.66 22014021

[2] Hazin R Ibrahimi O Hazin M Kimyai-Asadi A. Stevens-Johnson syndrome: pathogenesis, diagnosis, and management. *Ann Med.* (2008) 40:129–38. 10.1080/07853890701753664 18293143

[3] Lerch M Mainetti C Terziroli Beretta-Piccoli B Harr T. Current perspectives on Stevens-Johnson syndrome and toxic epidermal necrolysis. *Clin Rev Allergy Immunol.* (2018) 54:147–76. 10.1007/s12016-017-8654-z 29188475

[4] Bohigian G. *The history of Stevens-Johnson Syndrome and A Case Study.* St Louis: Center for History of Medicine at Washington University School of Medicine (2015). 10 p.

[5] University of Utah Burn Center. *Toxic Epidermal Necrolysis Syndrome (TEN) & Stevens-Johnson Syndrome (SJS).* (2021). Available online at: https://healthcare.utah.edu/burncenter/conditions-treatment/epidermal-necrolysis-syndrome-stevens-johnson-syndrome.php (accessed January 23, 2022).

[6] Bastuji-Garin S Fouchard N Bertocchi M Roujeau J Revuz J Wolkenstein P. SCORTEN: a severity-of-illness score for toxic epidermal necrolysis. *J Invest Dermatol.* (2000) 115:149–53. 10.1046/j.1523-1747.2000.00061.x 10951229

[7] Noe M Rosenbach M Hubbard R Mostaghimi A Cardones A Chen J Development and validation of a risk prediction model for in-hospital mortality among patients with stevens-johnson syndrome/toxic epidermal necrolysis—ABCD-10. *JAMA Dermatol.* (2019) 155:448–54. 10.1001/jamadermatol.2019.0998 30840032 PMC6459085

[8] Duplisea M Roberson M Chrisco L Strassle P Williams F Ziemer C. Performance of ABCD-10 and SCORTEN mortality prediction models in a cohort of patients with Stevens-Johnson syndrome/toxic epidermal necrolysis. *J Am Acad Dermatol.* (2021) 85:873–7. 10.1016/j.jaad.2021.04.082 33940101

[9] Koh H Fook-Chong S Lee H. Assessment and comparison of performance of ABCD-10 and SCORTEN in prognostication of epidermal necrolysis. *JAMA Dermatol.* (2020) 156:1294–9. 10.1001/jamadermatol.2020.3654 33084873 PMC7578915

[10] Jiang F Jiang Y Zhi H Dong Y Li H Ma S Artificial intelligence in healthcare: past, present and future. *Stroke Vasc Neurol.* (2017) 2:230–43. 10.1136/svn-2017-000101 29507784 PMC5829945

[11] Breiman L. *Random Forests.* Berkeley, CA: Statistics Department University of California (2001).

[12] Asaoka R Hirasawa K Iwase A Fujino Y Murata H Shoji N Validating the usefulness of the “Random Forests” classifier to diagnose early glaucoma with optical coherence tomography. *Am J Ophthalmol.* (2017) 174:95–103. 10.1016/j.ajo.2016.11.001 27836484

[13] Ferris L Harkes J Gilbert B Winger D Golubets K Akilov O Computer-aided classification of melanocytic lesions using dermoscopic images. *J Am Acad Dermatol.* (2015) 73:769–76. 10.1016/j.jaad.2015.07.028 26386631

[14] Dai B Chen R Zhu S Zhang W. Using random forest algorithm for breast cancer diagnosis. *Proceedings of the 2018 International Symposium on Computer, Consumer and Control (IS3C).* Taichung: IEEE (2018). p. 449–52. 10.1109/IS3C.2018.00119

[15] Fernandez-Lozano C Hervella P Mato-Abad V Rodríguez-Yáñez M Suárez-Garaboa S López-Dequidt I Random forest-based prediction of stroke outcome. *Sci Rep.* (2021) 11:10071. 10.1038/s41598-021-89434-7 33980906 PMC8115135

[16] Hanley J McNeil B. The meaning and use of the area under a receiver operating characteristic (ROC) Curve. *Radiology.* (1982) 143:29–36. *edit. 10.1148/radiology.143.1.7063747 7063747

[17] Retrouvey H Chan J Shahrokhi S. Comparison of two-dimensional methods versus three-dimensional scanning systems in the assessment of total body surface area estimation in burn patients. *Burns.* (2018) 44:195–200. 10.1016/j.burns.2017.07.003 28797577

[18] Dobry A Himed S Waters M Kaffenberger B. Scoring assessments in Stevens-Johnson syndrome and toxic epidermal necrolysis. *Front Med.* (2022) 9:883121. 10.3389/fmed.2022.883121 35783656 PMC9245022

[19] Guégan S Bastuji-Garin S Poszepczynska-Guigné E Roujeau J Revuz J. Performance of the SCORTEN during the first five days of hospitalization to predict the prognosis of epidermal necrolysis. *J Invest Dermatol.* (2006) 126:272–6. 10.1038/sj.jid.5700068 16374461

[20] Suo H Jiang B Sun X Dong J Alamgir M Guan X Comparing the accuracy of ABCD-10 and SCORTEN in predicting the in-hospital mortality of Stevens-Johnson Syndrome/Toxic epidermal necrolysis: a multi-institutional study from Central China. *Dermatology.* (2022) 238:736–44. 10.1159/000520494 34875648

[21] Koh H Fook-Chong S Lee H. Improvement of mortality prognostication in patients with epidermal necrolysis: the role of novel inflammatory markers and proposed revision of SCORTEN (Re-SCORTEN). *JAMA Dermatol.* (2022) 158:160–6. 10.1001/jamadermatol.2021.5119 34935871 PMC8696686

